# *In situ* procedure for high-efficiency computational modeling of atrial fibrillation reflecting personal anatomy, fiber orientation, fibrosis, and electrophysiology

**DOI:** 10.1038/s41598-020-59372-x

**Published:** 2020-02-12

**Authors:** Byounghyun Lim, Jaehyeok Kim, Minki Hwang, Jun-Seop Song, Jung Ki Lee, Hee-Tae Yu, Tae-Hoon Kim, Jae-Sun Uhm, Boyoung Joung, Moon-Hyung Lee, Hui-Nam Pak

**Affiliations:** Yonsei University Health System, Seoul, Republic of Korea

**Keywords:** Atrial fibrillation, Interventional cardiology

## Abstract

We previously reported the feasibility and efficacy of a simulation-guided clinical catheter ablation of atrial fibrillation (AF) in an in-silico AF model. We developed a highly efficient realistic AF model reflecting the patient endocardial voltage and local conduction and tested its clinical feasibility. We acquired > 500 endocardial bipolar electrograms during right atrial pacing at the beginning of the AF ablation procedures. Based on the clinical bipolar electrograms, we generated simulated voltage maps by applying fibrosis and local activation maps adjusted for the fiber orientation. The software’s accuracy (CUVIA2.5) was retrospectively tested in 17 patients and feasibility prospectively in 10 during clinical AF ablation. Results: We found excellent correlations between the clinical and simulated voltage maps (R = 0.933, p < 0.001) and clinical and virtual local conduction (R = 0.958, p < 0.001). The proportion of virtual local fibrosis was 15.4, 22.2, and 36.9% in the paroxysmal AF, persistent AF, and post-pulmonary vein isolation (PVI) states, respectively. The reconstructed virtual bipolar electrogram exhibited a relatively good similarities of morphology to the local clinical bipolar electrogram (R = 0.60 ± 0.08, p < 0.001). Feasibility testing revealed an *in situ* procedural computing time from the clinical data acquisition to wave-dynamics analyses of 48.2 ± 4.9 min. All virtual analyses were successfully achieved during clinical PVI procedures. We developed a highly efficient, realistic, *in situ* procedural simulation model reflective of individual anatomy, fiber orientation, fibrosis, and electrophysiology that can be applied during AF ablation.

## Introduction

Catheter ablation (CA) is an effective approach for rhythm control management of atrial fibrillation (AF)^1,2^. However, the recurrence rate after AF ablation procedures is still substantial^3^. Although pulmonary vein isolation (PVI) is a well-established target of AF ablation, extra-pulmonary vein (PV) foci or drivers maintain AF in some patients, and extra-PV foci are more commonly found in AF patients with significant left atrial (LA) remodeling^4,5^. The Substrate and Trigger Ablation for Reduction of Atrial Fibrillation trial part 2 (STAR AF2) demonstrated that an empirical extra-PV ablation did not improve the rhythm outcome compared with a circumferential PVI alone in patients with persistent AF (PeAF)^6^. However, the one-year recurrence rate was higher than 40% regardless of any additional extra-PV ablation after the PVI, and the outcome of the invasive interventional catheter procedure was not adequate. Therefore, an innovative mapping technology to identify the core target of AF is needed in AF catheter ablation (AFCA).

Simulation is a very useful computer-aided method for identifying appropriate intervention targets. We recently reported the feasibility of a simulation-guided PeAF ablation by applying a personalized heart computed tomography (CT) image-integrated AF simulation^7,8^. To further this method, we developed a more realistic AF simulation reflective of personalized anatomy, fiber orientation, fibrosis, and electrophysiology. We upgraded our software (CUVIA2.5), which applies the clinically acquired voltage and activation data during the AF ablation procedure and can provide personalized AF wave-dynamics information to the operator. The purpose of this study was to validate the accuracy of the CUVIA2.5 by a retrospective clinical study and to test the feasibility of the realistic modeling-guided AF ablation in a prospective clinical study.

## Methods

The study protocol was approved by the Institutional Review Board of Severance Cardiovascular Hospital, Yonsei University Health System, and adhered to the Declaration of Helsinki. This study is registered at Clinicaltrials.gov (NCT 02171364). All subjects provided written informed consent for the use of their cardiac CT images and clinical electrophysiological mapping data for the computational modeling studies.

### 3D computational model of the left atrium

**Figure 1:**
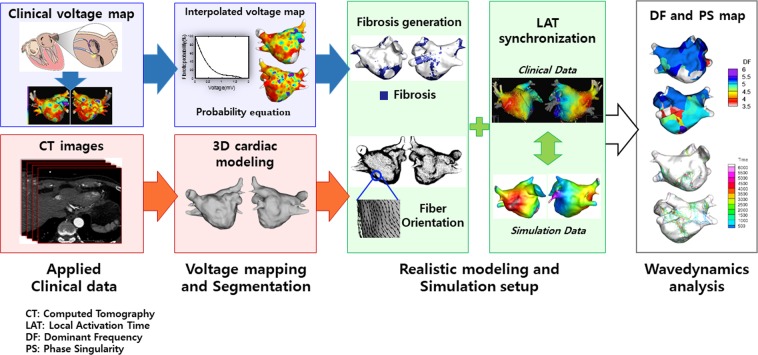
Study protocol. Outline of the realistic modeling reflecting the patient’s anatomy, fiber orientation, and fibrosis.

Ionic currents in each cell were determined using the human atrial myocyte model developed by Courtemanche *et al*.^9^. For remodeling of ion currents of AF, I_K1_ and I_NCX_ were increased by 100% and 40%, and I_Na_, I_to_, I_CaL_, and I_Kur_ were decreased by 10%, 70%, 50%, and 50%, respectively^10,11^. We developed our GUI software (CUVIA ver. 2.5, Model: SH01; Laonmed Inc., Seoul, Korea)^7^ so that it could implement not only virtual AF induction and the AF wave-dynamics by phase singularities (PS) and by the dominant frequency (DF), but also fiber orientation and fibrosis formation, onto the LA surface. The system can also generate a realistic 3D *in silico* AF model from a patient’s clinical data. Figure 1 comprises an outline of modeling progression. We generated 3D modeling-integrated CT images of the LA^12,13^. Then, a 3D mesh was generated with 400,000~500,000 nodes and was refined as a triangular type; the mean distance between adjacent nodes was 235.1 ± 32.1 μm.

### Acquisition of clinical electro-anatomical maps

Clinical electro-anatomical maps were acquired to define the tissue characteristics of the model. We collected clinical data that included the bipolar electrograms recorded from > 500 points on the atrial surface to develop interpolated voltage data. The clinical electrogram data comprised sequential recordings during a paced rhythm, with a cycle length of 500 ms. The locations of the 3D model, obtained from an EnSite NavX system (Abbott Inc., Lake Bluff, IL USA), were matched with the coordinates of the clinical map after merging with the patient’s heart CT images. The voltage values and coordinates of each clinical catheter point were stored.

### Alignment of electro-anatomical maps onto CT derived mesh models

Registration of the electro-anatomical maps onto the CT models involved the four following steps: (1) geometry, (2) trimming, (3) field scaling, and (4) alignment. Each step was performed manually by aligning anatomical features. First, in the geometry step, an electro-anatomical map was created using a circular mapping catheter based on CT images. After the electro-anatomical map was created, the trimming step undertaken. During this stage, any artifact caused by the patient’s breathing was removed, and the PV and LA appendage (LAA) sites were divided based on the ostial position. Next, field scaling was applied to optimized the electro-anatomical map based on interelectrode spacing and to match the size thereof with that of the CT image as close as possible. In the alignment step, alignment points were based on a precisely defined ostium. Upon applying the alignment points in the same position, registration was completed. The alignment comprised rigid coordinate transformation. The mean registration error was estimated as 1.82 ± 1.25 mm. In this case, the catheter points representing electro-anatomical map and the segmented CT image of the LA were integrated.

### Interpolation of the electro-anatomical map values on the mesh

**Figure 2:**
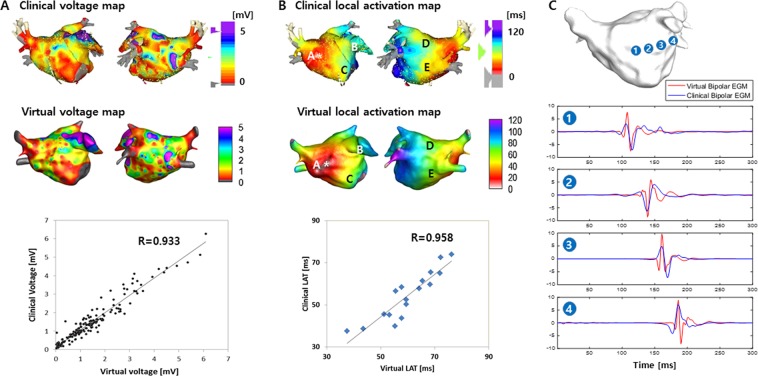
Comparison of the clinical and virtual modeling. (**A**) Comparison of the clinical and virtual voltage, (**B**) Comparison of the clinical and virtual local activation time (LAT), (**C**) Normalized signal between the clinical and virtual electrograms.

Virtual voltage data were created by an interpolation of the clinical voltage mapping. We used the inverse distance weighting (IDW) method for signal interpolation^14^. We collected the data within a radius of 10 mm from the point where we wanted to interpolate the data and interpolated it using IDW to do so. An interpolated voltage map generated by our customized software is shown in Fig. 2A, which is similar to that of the NavX system.

### Definition of fiber orientation from atlases

We accounted for fiber orientations using an atlas based mesh of each patient’s geometry^15,16^ and generated high-speed, high-density, and whole-chamber AF mapping using personalized electrophysiologic mapping data. The GPU fiber tracking process involved two stages: tracking and visualization. Fiber tracking was a parallel task, since the same algorithm was executed for each seed point, independent from each other, making it effective to use the GPU system. In the fiber orientation map, the difference in conduction according to the orientation was realized through the fiber tracking method. A vector along the myocardial fiber direction could be created at each point of the heart. The conductivity in the direction perpendicular to the vector was smaller than the conductivity in the vector direction. We adjusted the fiber orientation based on the clinical LAT map.

### Spatial distribution of fibrosis based on clinical voltage map

The locations of fibrotic areas were determined based on a clinically acquired bipolar voltage map. First, bipolar voltage data from the clinically acquired map were interpolated onto the computational nodes on the 3D in silico atrial model. To determine fibrosis status (yes/no) for each node, we used the following nonlinear relationship between the bipolar voltage and probability of fibrosis^17^:

1 $${{P}}_{{\rm{fibrosis}}}=\{\begin{array}{rl}1, & {\rm{X}} < 0\\ -40.0{X}^{3}+155{X}^{2}-206X+99.8 & 0\le {\rm{X}}\le 1.74\\ 0, & 1.74 < {\rm{X}}\end{array}$$

where *P*_fibrosis_ is the probability that there is fibrosis at a given node and X is the bipolar voltage at that node in the range of 0 to 1.74 mV. If X is greater than 1.74 mV, *P*_fibrosis_ converges to zero. This was developed by comparing the predicted percentage of fibrosis across the 3D atrial model with the pre- and post-ablation fibrosis data. For each node, the probability of fibrosis calculated based on the clinically acquired bipolar voltage data was compared against a random number between 0 and 1.

### Definition of base electrophysiological parameters of fibrotic and non-fibrotic tissue

Using a model that reflects the structural orientation of the heart and fiber orientation, we created a model similar to the clinical local activation data as shown in Fig. 1. The conductivity of the model was applied at 0.1264 S/m (non-fibrotic longitudinal cell), 0.0546 S/m (fibrotic longitudinal cell), 0.0252 S/m (non-fibrotic transverse cell), and 0.0068 S/m (fibrotic transverse cell)^18^. If the random number was below the calculated probability of fibrosis, the node was considered to have a positive fibrosis status. Compared with normal cells, the ion current of the fibrosis cell, that is, the inward rectifier potassium current (I_K1_), L-type calcium current (I_CaL_), and sodium current (I_Na_), decreased by 50%, 50%, and 40%, respectively^18^. We defined the longitudinal CV as that in the same direction as the vector and the transversal CV as that in the perpendicular direction to the vector.

### Customized tuning of parameters according to clinical conduction velocity and LAT synchronization

After obtaining the clinical local activation time (LAT) data and applying the fiber orientation and fibrosis on the 3D LA model, we conducted a synchronization between the clinical and virtual LAT maps to determine the conduction velocity (CV) as a preliminary simulation (Fig. 1). We modulated diffusion coefficients of the patient-specific atrial model by matching the CV obtained from the simulation to the clinical CV. We compared the virtual LAT and clinical LAT maps by measuring CV at five different points (Fig. 2B). To calculate the CVs of the clinical and simulation data, the conduction distances from the earliest activation site (EAS) near Bachmann’s bundle^7^ to five different activation points were measured on the surface of the simulation model and were divided by the time difference as previously described^19^. We adjusted the virtual conduction time by modulating the diffusion coefficient of the model. We then performed a comparison of the clinical and virtual LAT maps. In this study, the virtual CV was adjusted to the clinical LAT map after fibrosis was applied. We adjusted the longitudinal and transverse diffusion coefficients at the same rate. The calculated longitudinal CV was 0.707 ± 0.029 m/s, and the transverse CV was 0.486 ± 0.010 m/s.

### Virtual AF induction and analyses of PS and DF

Ramp pacing stimulation, which was performed with cycle lengths from 200 to 120 ms, was applied near Bachmann’s bundle^7^. The overall pacing duration was 6,530 ms. In order to establish the criteria of the action potential duration (APD) and CV in this model, the APD and CV were determined using the average data from the clinical Yonsei AF ablation cohort (n = 3,030). The effective refractory period and CV values measured in the clinical database were 217.3 ± 71.4 ms and 0.45 ± 0.24 m/s CV, respectively^7,19^. The APD_90_ and CV values measured in the simulation modeling were 206.5 ± 6.8 ms and 0.50 ± 0.06 m/s, respectively. When AF was successfully induced, we analyzed the wave-dynamics of the PS and DF for 6s^7^. The DF was defined as the frequency with the highest power. The power spectral density was obtained by a Fourier transformation of the virtual action potential of each node. We calculated the DF values for all nodes of the 3D LA model. The peak point of the DF map and the highest 5% DF map were generated. The highest 5% DF areas were defined by nodes that showed the top 5% DF values. A PS trajectory map on the atrial surface was also generated. The PS was defined at the point where the phase was undetermined^20,21^. We sampled the 6-s data for a single PS calculation in our previous study^7^. We then used the location-centric method^21^ that we previously developed for identifying the phase singularity points with a high computational speed and accuracy. We previously validated our method by comparing it with the Iyer-Gray method. In our previous study of electrophysiological rotor ablation in an in-silico modeling^22^, the mother rotor was defined as the rotor with a tip that remains within a circle of the diameter that is half the wavelength of the cardiac wave for longer than 5 s. The wavelength was determined for the linear wave generated by a line while pacing with a 600-ms cycle length before the initiation of reentry. The rotor tip was defined as the PS point of the rotor.

### Numerical solution and accelerated computing

The finite difference method was used for numerical modeling of the atrium and solution in the triangular type mesh^23^ (Supplementary Fig. S1A). Most mathematical functions were performed with the compute unified device architecture (CUDA) system, based on C and C +  + languages, and parallel transaction was conducted using a graphic processing unit (GPU). We used the CUDA system to perform a parallel computing and calculated the results at very high speeds by grouping register memories (Supplementary Fig. S1B). Since the complex exponential or logarithm calculations were evaluated at a high cost in the computation, we used a lookup table for faster computation speeds (Supplementary Fig. S1C). We calculated the extracellular potential for the two electrodes of the bipolar catheter. The unipolar electrograms at different points of the atria surface under conditions of uniform intracellular anisotropic resistivity were simulated, as previously described^24^. The extracellular potential (ϕ_e_) was given by the following equation:

2 $${\O }_{{e}}({r})=-\frac{1}{4{\pi }}\frac{{{\rm{\sigma }}}_{{i}}}{{{\rm{\sigma }}}_{{e}}}\iiint \nabla {{V}}_{{m}}({r}{\prime} )\cdot \nabla [\frac{1}{{r}{\prime} -{r}}]{dv}$$

where ∇V_m_ is the spatial gradient of the transmembrane potential V_m_, σ_i_ the intracellular conductivity, σ_e_ the extracellular conductivity, r the distance from the source point (x, y, z) to the measuring point (x’, y’, z’), and dv the differential volume. The bipolar electrograms were calculated by subtracting two adjacent unipolar electrograms spaced 1 mm apart. The time step was adaptively varied between 0.01 and 0.1 ms, and we opted for double precision for a higher numerical accuracy using the operator splitter method^10,25^.

### Accuracy validation of retrospective simulation

To validate the realistic AF modeling generated by the CUVIA2.5, we investigated the correlations between the pre-acquired clinical voltage mapping data and retrospective simulated mapping results (both during a pacing state) in 17 patients who underwent AF ablation (70.6% male, 60.4 ± 8.4 years old, 58.8% PeAF, Table 1). Using the clinical bipolar electrogram data (from 7 PeAF, 5 paroxysmal AF [PAF], and 5 post-PVI PAF patients), we integrated patient-specific heart CT imaging of the LA, spatiotemporal locations in bipolar electrograms, degree of fibrosis based on the clinical voltage maps, and fiber orientation based on clinical activation time. Morphological similarity of the bipolar electrograms was calculated by the mean of the inner product between the normalized clinical and simulated electrogram signals by the following equation^26,27^:

3 $$\frac{{EG}{{M}}_{{Cli}}\cdot {EG}{{M}}_{{Vir}}}{\Vert {EG}{{M}}_{{Cli}}\Vert \,\Vert {EG}{{M}}_{{Vir}}\Vert }$$

where EGM_Cli_ is the clinical bipolar vector and EGM_Vir_ the virtual bipolar vector. Figure 2C shows an example of a normalized signal between a clinical and virtual electrogram. We used these data to generate realistic AF modeling. All simulation data were calculated by CUVIA software version 2.5 (Laonmed Inc., Seoul, Korea). We compared the similarities between the clinical and simulated voltage maps and that between the clinical and virtual LAT maps.

**Table 1:** Patient characteristics.

	Retrospective accuracy study (17 patients)	Prospective feasibility study (10 patients)
Age, years (Mean ± SD)	60.4 ± 8.4	55.2 ± 9.5
>65 years old	7 (41.2%)	1 (10%)
<65 years old	10 (58.8%)	9 (90%)
Gender		
Male	12 (70.6%)	4 (40%)
Female	5 (29.4%)	6 (60%)
Persistent AF	10 (58.8%)	10 (100%)
Heart failure	3 (17.7%)	3 (30%)
Hypertension	10 (58.8%)	4 (40%)
Diabetes	1 (5.9%)	3 (30%)
Previous stroke	0 (0%)	0 (0%)
Previous TIA *	0 (0%)	0 (0%)
Vascular disease	0 (0%)	0 (0%)
Left atrium dimension	40.5 ± 4.9 mm	46.1 ± 5.6 mm
Ejection fraction	60.9 ± 11.6%	60.4 ± 4.8%
E/Em †	10.4 ± 3.3	10.2 ± 4.3

^*^TIA, transient ischemic attack; † E/Em, the ratio of the early diastolic mitral inflow velocity (E) to the early diastolic mitral annular velocity (Em).

### Prospective clinical feasibility during the AF ablation procedure

To test the clinical feasibility of a personalized realistic modeling of AF, we prospectively tested the *in silico* modeling in 10 patients (40% male, 55.2 ± 9.5 years old, all PeAF) during AF ablation procedures. At the beginning of the procedure, an on-site procedure team acquired the LA bipolar electrograms at 500~1000 points after internal cardioversion (5~30 J) and sent the spatiotemporal data to the core lab by e-mail or via the internal network after integration of the heart CT imaging (100~300 Mb in size). The operators started the circumferential PVI as soon as the bipolar electrogram data were exported. The core lab team conducted the simulation study using the patient data obtained from the procedure team. After analysis of the wave-dynamics with PS and DF maps, the analyzed results were sent back to the on-site procedure team by e-mail or the internal network, and the calculation time of the core lab was monitored to determine whether the simulation results could be provided within a routine circumferential PVI procedure.

### Statistical analysis

Data are expressed as the mean ± standard deviation. Data for the retrospective and prospective feasibility simulation study were compared using a Pearson’s correlation. A p-value < 0.05 was considered statistically significant. The statistical analyses were performed using SPSS software for Windows (version 20.0, Statistical Package for Social Sciences, Chicago, IL, USA).

## Results

### Comparison of the clinical electrogram-based map and virtual modeling

**Figure 3:**
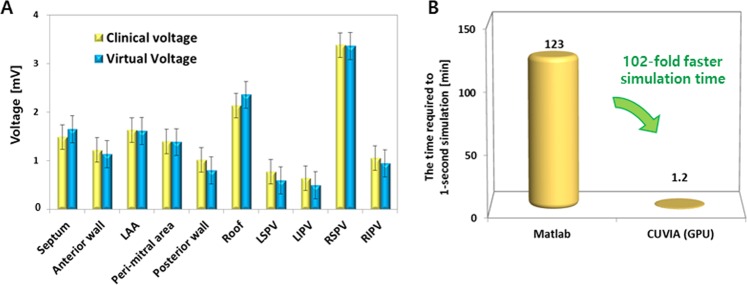
(**A**) Regional voltage of the clinical and virtual data, (**B**) The calculation time for a realistic AF modeling using CUVIA and Matlab software under a condition of 500,000 nodes.

We compared the endocardially-acquired clinical bipolar voltage with the simulated voltage map in 17 patients, as shown in Fig. 2A. We observed an excellent correlation between the clinical data and simulation models (R = 0.933, p < 0.001, Fig. 2A and Table 2). In a regional sub-analysis, the LA was divided into 10 areas (Supplementary Fig. S1D), and the regional clinical voltage and virtual voltage were evaluated (Fig. 3A). There was a high correlation between the clinical voltage and virtual voltage in each region (Table 2). Although the R-value was lowest in the left inferior PV area (R = 0.727), the correlation was still significant (p < 0.001). To compare the local CVs in the clinical map and simulation map, we measured the conduction times from the earliest activation site (A, asterisk in Fig. 2B) to the other areas (LAA, mitral annulus 12 o’clock area, LA roof, and bottom of the left inferior pulmonary vein; b–e in Fig. 2B). The clinical and virtual LAT maps were similar, and the local conduction times measured were significantly correlated (R = 0.958, p < 0.001, Table 3, Fig. 2B). In the morphological analysis, the reconstructed virtual bipolar electrograms exhibited a relatively good similarities of morphology (R = 0.60 ± 0.08, p < 0.001) to the local clinical bipolar electrograms (Fig. 2C).

**Table 2:** Mean voltage of 10 LA segments and the correlation between the clinical and simulated voltage map.

	Clinical voltage (mV)	Simulated voltage (mV)	R value	P value	RMSE (%RMSE)
Septum	1.49 ± 0.65	1.65 ± 0.60	0.940	<0.001	0.21 (12.9%)
Anterior wall	1.22 ± 0.40	1.13 ± 0.36	0.950	<0.001	0.16 (14.5%)
Left atrial appendage	1.63 ± 0.91	1.61 ± 0.77	0.956	<0.001	0.18 (11.1%)
Peri-mitral area	1.40 ± 0.85	1.39 ± 0.74	0.988	<0.001	0.27 (19.4%)
Posterior wall	1.01 ± 0.80	0.80 ± 0.81	0.943	<0.001	0.13 (15.8%)
Roof	2.14 ± 1.23	2.36 ± 1.33	0.985	<0.001	0.33 (13.9%)
Left superior PV	0.78 ± 0.67	0.60 ± 0.56	0.943	<0.001	0.19 (32.1%)
Left inferior PV	0.64 ± 0.47	0.50 ± 0.37	0.727	<0.001	0.18 (36.6%)
Right superior PV	3.38 ± 0.73	3.36 ± 1.34	0.942	<0.001	0.20 (5.9%)
Right inferior PV	1.06 ± 0.73	0.95 ± 0.50	0.953	<0.001	0.24 (25.1%)
Overall	1.48 ± 0.76	1.44 ± 0.83	0.933 ± 0.07	<0.001	

RMSE: Root mean square error, %RMSE: Percentage root mean square error.

**Table 3:** Comparison between the clinical and virtual local conduction times (CTs).

Patients	Clinical CT (ms)	Virtual CT (ms)	R value	P value	RMSE (%RMSE)
1	38.8 ± 7.6	43.5 ± 7.4	0.967	0.032	5.12 (11.8%)
2	37.5 ± 8.3	37.5 ± 10.6	0.999	0.001	2.35 (6.3%)
3	56.5 ± 30.1	55.5 ± 31.3	0.979	0.021	6.52 (11.7%)
4	40.0 ± 8.2	55.3 ± 12.6	0.999	0.001	15.90 (28.8%)
5	65.0 ± 20.9	72.0 ± 21.7	0.999	<0.001	7.11 (9.9%)
6	61.5 ± 22.4	65.5 ± 18.8	0.989	0.011	6.20 (9.5%)
7	57.8 ± 14.4	64.3 ± 21.7	0.972	0.028	10.61 (16.5%)
8	45.5 ± 12.8	51.3 ± 18.6	0.973	0.027	8.90 (17.4%)
9	59.8 ± 11.7	68.3 ± 13.9	0.906	0.094	10.37 (15.2%)
10	58.5 ± 13.6	57.8 ± 18.8	0.992	0.008	5.55 (9.6%)
11	72.5 ± 21.4	72.3 ± 21.5	0.993	0.007	2.50 (3.5%)
12	43.8 ± 15.2	57.8 ± 18.0	0.961	0.039	15.02 (26.0%)
13	52.5 ± 12.3	59.5 ± 16.3	0.644	0.356	14.40 (24.2%)
14	50.3 ± 16.4	59.9 ± 12.2	0.978	0.021	10.57 (17.8%)
15	74.0 ± 20.9	76.3 ± 20.7	0.973	0.027	5.32 (7.0%)
16	45.3 ± 10.2	53.3 ± 13.2	0.962	0.038	9.11 (17.1%)
17	65.5 ± 11.4	68.5 ± 11.9	0.999	<0.001	3.08 (4.5%)
Overall	54.4 ± 11.1	59.9 ± 10.0	0.958 ± 0.08	<0.001	

RMSE: Root mean square error, %RMSE: Percentage root mean square error.

### Degree of fibrosis in the virtual AF map

We generated an interpolated voltage map by applying fibrosis, and the degree of fibrosis was calculated using the clinical local voltage ‘X’ in Eq. 1^18^. The calculated fibrosis was 15.4% in patients with PAF (n = 5), 22.2% in patients with PeAF (n = 7), and 36.9% during the post-PVI state (n = 5). For the analysis of the spatial relationship between the fibrosis and PS or DF, we calculated the proportion of fibrotic nodes, number of PS occurrences, and DF values in the 10 segmented regions presented in Supplementary Fig. S1D. The regional number of PS had a good correlation to the degree of fibrosis (R = 0.87, p < 0.001). High DF areas were generally localized at the periphery of fibrosis and had a poor regional correlation to the fibrotic area (R = 0.38, p < 0.001, Supplementary Fig. S2 and Supplementary Table S1). The degree of atrial fibrosis affected the AF wave-dynamics.

### Processing time for AF modeling

We performed the simulation using the GPU-based parallel calculation system for a highly efficient computation speed. With our CUDA system, it took about 1.2 minutes to run a 1-second simulation in a LA model with 400,000~500,000 nodes, and the computational time increased by 36 sec with every 10,000 additional nodes. The system can be simulated 102-fold faster than the previous calculating method using Matlab under the same condition (Fig. 3B). During the AFCA procedures in the 10 patients with PeAF, we acquired a clinical endocardial voltage map and LAT map (NavX, Abbott Inc. U.S.A.) and conducted a realistic simulation based on the clinically acquired data. The calculated time from the clinical electrogram data extraction to the report of the virtual DF and PS maps was 48.2 ± 4.9 min. After extraction of the clinical data, the operator immediately started the PV isolation procedure and received a report of the patient’s virtual DF map and PS map before the PVI procedure was complete, in all cases (100%). We present here a representative case of computational modeling in a patient with persistent AF (Supplementary Fig. S3). In this case, the computational time was 41 min, and the highest DF site was close to the posterior aspect of the left side of the PV in spite of a heterogeneous distribution of the DF and PS. Sustaining AF was successfully terminated during the left-side CPVI, and we did not conduct any extra-PV LA ablation in this patient with persistent AF.

## Discussion

In this study, we generated a highly efficient realistic simulation modeling of AF, which could be applied to the clinical AF ablation procedure. We proved the accuracy of the model by the similarity of virtual and clinical electrograms and the association of fibrosis and wave-dynamic parameters. At the beginning of the AF ablation procedure, we acquired bipolar electrograms with > 500 points during RA pacing and sent a file containing the electrograms and their spatial location data to the core lab by e-mail or via the network. A patient-specific realistic AF modeling, reflecting the degree of fibrosis, activation pattern, and fiber orientation, was performed, and virtual AF was induced based on a highly efficient computing algorithm (72-s calculation times for 1-s AF simulation). We conducted additional AF wave-dynamics analyses, and the results were reported to the operator within 50 min during the PVI procedure. To the best of knowledge, this study is the first study to verify the clinical applicability of highly efficient realistic computational modeling of AF in reflection of a patient’s atrial anatomy, fiber orientation, fibrosis, and electrophysiology in a retrospective and prospective clinical application.

AF ablation reduces the heart failure mortality^28^ and stroke risk^29^ and improves the cognitive function^30^ and renal function^31^. However, AFCA is a time consuming and challenging procedure with a substantial recurrence rate, especially in patients with PeAF. Because the PVI is the cornerstone of AFCA, we conducted a circumferential PVI (CPVI) in every patient during the AFCA procedure. However, the ablation of non-PV triggers is also important to improve the rhythm outcome of AFCA. Therefore, we acquired bipolar voltage maps at the beginning of the procedure and ran a simulation study to detect any extra-PV AF drivers during the CPVI procedure^6,32^. An empirical extra-PV ablation is partially effective in some patients with PeAF, but a prospective randomized clinical trial failed to prove its usefulness^6^. Moreover, an empirical extra-PV ablation may increase the LA pressure and has the risk of causing a stiff LA syndrome. Clinical investigators have thus been tracing personalized extra-PV AF rotors or drivers over the last 7 years^33,34^. Rotors can exist in AF but not as a stationary, single, mother rotor, as Jalife originally defined in ventricular fibrillation^35^. We recently demonstrated the effectiveness of virtual ablation targeting AF spiral wave reentries represented by a high DF area^22^, but the spatiotemporal stability of the high DF area changes depending on the CV^8^ in simulation modeling studies. Thus, it is not clear whether burning of non-stationary phantom targets results in consistent outcomes in patients with AF. Instead, a more accurate, sophisticated, and fast mapping technique that reflects patient-specific anatomy, fiber orientation, fibrosis, and electrophysiology, which can affect AF wave-dynamics, is required. In this study, the number of PS was higher in areas with fibrosis, which was consistent with the previous reports^36^. However, the DF area was localized to the periphery of the fibrotic area with a poor correlation, consistent to Koduri’s report^37^.

The computational cardiac simulation study has a growing role and allows the non-invasive identification of atrial reentries in AF^38^. The contemporary clinical 3D mapping system has enabled electro-anatomical mapping of the atrium in detail after heart image integration, but it is not possible for identifying non-stationary AF drivers or spiral reentries of sustained AF by a point-to-point catheter mapping. Although the entire chamber AF mapping technique, such as focal impulse and rotor mapping (FIRM)^33^ and panoramic mapping^34^, has been developed, there is a limit to the spatial resolution of the AF map. In recent years, high-performance modeling reflecting the MRI late gadolinium-enhanced fibrosis, fiber orientation, and atrial thickness has been developed and has been clinically applied (Supplementary Table S1)^39–42^. Using this sophisticated simulation modeling, high-density entire chamber mapping of AF, reflecting personalized anatomy, fiber orientation, and fibrosis is possible. Nevertheless, evidence of the relationship between MRI-characterized scar and real histological fibrosis has not been proven except in ischemic cardiomyopathy, and bilayer atrial modeling is still best for the application of the atrial wall thickness^40^. Despite the relatively well-developed AF modeling technology, the highest hurdle in applying the simulation to clinical practice is the long calculation time. In this study, high-speed, high-density, whole-chamber AF mapping using personalized electrophysiologic mapping data was made possible by speeding up the computing calculation, and the simulation results were directly applied to the AF ablation procedure. By using this *in situ* procedural high-efficiency modeling of AF, we expect to be able to increase the ablation success rate of PeAF patients by detecting and ablating more precise, reproducible, and consistent extra-PV AF drivers.

Although this study considered patient-specific atrial anatomy, fiber orientation, fibrosis, and electrophysiology in a realistic *in silico* modeling of AF, the model was a monolayer design. An atrial thickness variation could affect the wave propagation in the area of an abrupt change in the fiber orientation, but the wave propagation pattern of the monolayer model is reported to be similar to that of the multilayer model^43^. Moreover, we could not reflect the epicardial conduction pattern by the endocardially-acquired clinical voltage and local activation pattern. A reflection of the accurate atrial wall thickness may improve the elaborate wave-dynamics analyses as well as be applicable to radiofrequency energy titration during clinical AF ablation. We did not analyze the AF after multiple site stimulation. Although the AF dynamics can be affected by the induction site^44^, the spatial distribution of the AF wave dynamic parameters does not significantly differ from spontaneously induced AF, regardless of the induction site^45^. The clinical relevance of the study was limited due to the lack of clinical data on simulation-guided ablation, and a well-designed clinical trial is warranted. The DF and PS might be insufficient to characterize the complex AF wave-dynamics and to guide ablation, because the fibrillation process is complex and driven by focal sources or a pure multiple wavelet mechanism. The validity of the PS in the identification of reentrant activity is still controversial^46^. Although the registration between the electroanatomical map and the CT model was performed by an experienced technician, we cannot exclude any registration error^47^ during the clinical mapping process that may have had an impact on the accuracy of the model. In a recent study, the time required for the PVI was shortened by attempting ablation with high-power, short-duration radiofrequency energy. Therefore, further shortening of the simulation computation time is required. The current realistic AF model was simulated using invasive clinical data, and simulation using less invasive clinical data is necessary for the use of this model in a wider range of AF patients.

## Conclusion

We developed a realistic simulation model for AF reflecting personal anatomy, fiber orientation, fibrosis, and electrophysiology using a bipolar electrogram-generated map acquired during the AF ablation procedure. This high-performance model would enable a patient-specific virtual intervention or virtual drug therapy after further validation.

## Supplementary information

Supplementary information.
