# Supplementary material for: In situ procedure for high-efficiency computational modeling of atrial fibrillation reflecting personal anatomy, fiber orientation, fibrosis, and electrophysiology
Source: Sci Rep. 2020 Feb 12;10:2417. doi: 10.1038/s41598-020-59372-x (PMC7016008; doi:10.1038/s41598-020-59372-x)

Supplementary information

In situ procedure for high-efficiency computational modeling of atrial fibrillation reflecting personal anatomy, fiber orientation, fibrosis, and electrophysiology

Byounghyun Lim, Jaehyeok Kim, Minki Hwang, Jun-Seop Song, Jung Ki Lee, Hee-Tae Yu, Tae-Hoon Kim, Jae-Sun Uhm, Boyoung Joung, Moon-Hyung Lee, Hui-Nam Pak*

Yonsei University Health System, Seoul, Republic of Korea

Table S1. Proportion of fibrotic cell, number of PS occurrences, and DF values in 10 segmented regions of LA

10 segmented regions of LA	Percentage of fibrotic cell [%]	Percentage of PS occurrence cell [%]	DF [Hz]
1. Septum	16.82	52.71	7.79
2. Anterior wall	1.86	6.04	8.15
3. LAA	1.46	8.17	8.30
4. Peri-mitral area	27.87	78.51	8.38
5. Posterior inf. wall	13.22	57.08	8.41
6. Posterior wall	2.80	10.53	7.75
7. Left upper PV	13.72	20.55	8.12
8. Left lower PV	5.77	4.44	7.88
9. Right upper PV	18.34	26.89	8.20
10. Right lower PV	4.95	2.66	7.88

Figure S1. Numerical solution, accelerated computing, and segmented LA regions.

A. Applying effective Finite Difference Method for simulation

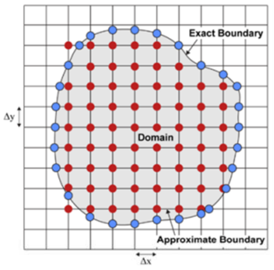

B. CUDA-based parallel computing system

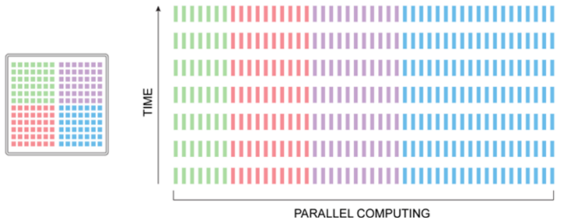

C. Voltage-based lookup table for pre-calculating high cost operation

Sequence	Indexing number	Indexing step	Content
0	00000000000000000	0.01	Value + (Indexing step x 00000000000000000)
1	00000000000000001	0.01	Value + (Indexing step x 00000000000000001)
…	…	…	…
2^{17}-2	11111111111111110	0.01	Value + (Indexing step x 11111111111111110)
2^{17}-1	11111111111111111	0.01	Value + (Indexing step x 11111111111111111)

D. 10 regions of LA

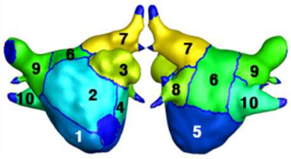

1.Septum
2.Anterior wall
3.Left atrial appendage
4.Peri-mitral area
5.Posterior wall
6.Roof
7.Left superior PV
8.Left inferior PV
9.Right superior PV
10.Right inferior PV

Figure S2. Relationship between fibrosis and PS and DF.

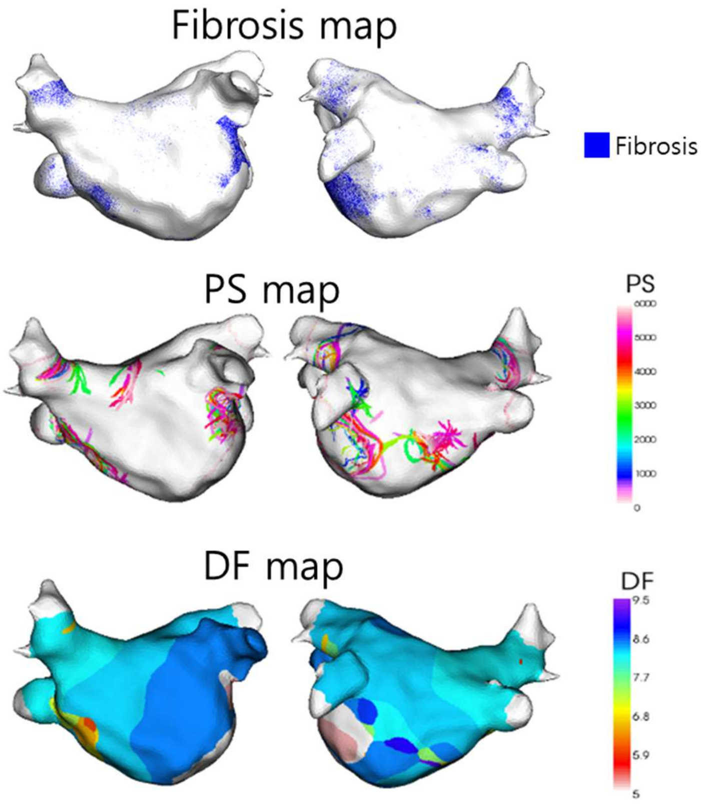

Figure S3. Representative case of computational modeling in patient with persistent AF.

A. Clinical and virtual LAT map, B. Clinical and virtual voltage map, C. Clinical ablation site, D. Virtual DF map for 6s, E. Virtual PS map for 6s, F. Action potential of the highest DF site.

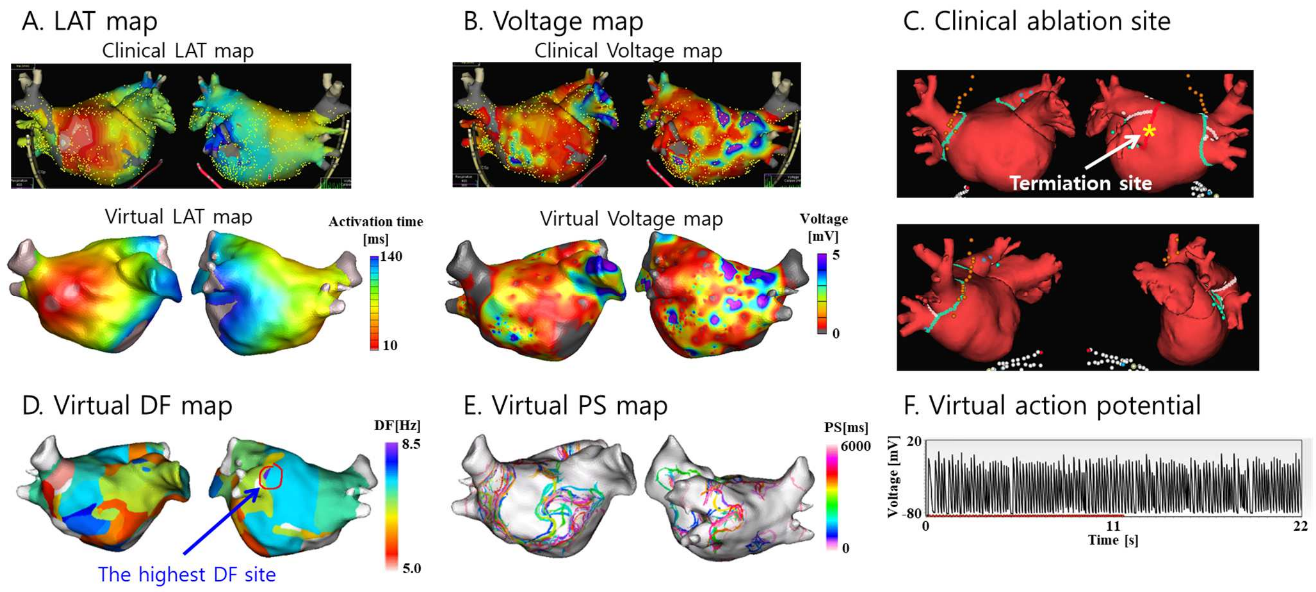

Supplement: Supplementary file 1 — Supplementary information. [file 41598_2020_59372_MOESM1_ESM.pdf]
